# Methods for improving thermal tolerance in military personnel prior to deployment

**DOI:** 10.1186/s40779-020-00287-z

**Published:** 2020-11-29

**Authors:** Edward Tom Ashworth, James David Cotter, Andrew Edward Kilding

**Affiliations:** 1 Sports Performance Research Institute New Zealand (SPRINZ), Auckland University of Technology, 17 Antares Place, Rosedale, Auckland, 0632 New Zealand; 2 School of Physical Education, Sport and Exercise Sciences, University of Otago, Dunedin, Otago 9016 New Zealand

**Keywords:** Heat acclimation, Thermoregulation, Heat illness, Physiology, Human, Conditioning, Military

## Abstract

Acute exposure to heat, such as that experienced by people arriving into a hotter or more humid environment, can compromise physical and cognitive performance as well as health. In military contexts heat stress is exacerbated by the combination of protective clothing, carried loads, and unique activity profiles, making them susceptible to heat illnesses. As the operational environment is dynamic and unpredictable, strategies to minimize the effects of heat should be planned and conducted prior to deployment. This review explores how heat acclimation (HA) prior to deployment may attenuate the effects of heat by initiating physiological and behavioural adaptations to more efficiently and effectively protect thermal homeostasis, thereby improving performance and reducing heat illness risk. HA usually requires access to heat chamber facilities and takes weeks to conduct, which can often make it impractical and infeasible, especially if there are other training requirements and expectations. Recent research in athletic populations has produced protocols that are more feasible and accessible by reducing the time taken to induce adaptations, as well as exploring new methods such as passive HA. These protocols use shorter HA periods or minimise additional training requirements respectively, while still invoking key physiological adaptations, such as lowered core temperature, reduced heart rate and increased sweat rate at a given intensity. For deployments of special units at short notice (< 1 day) it might be optimal to use heat re-acclimation to maintain an elevated baseline of heat tolerance for long periods in anticipation of such an event. Methods practical for military groups are yet to be fully understood, therefore further investigation into the effectiveness of HA methods is required to establish the most effective and feasible approach to implement them within military groups.

## Background

Military units deploying abroad are often exposed to challenging environments. One is heat stress, which presents as high ambient temperature, often augmented by high humidity, resulting in heat strain. Heat strain affects many physiological systems, compromising physical and potentially also cognitive performance [1–7]. Heat has impaired military performance for thousands of years, with ancient Greek and Roman reports highlighting the dangers of warfare in the heat, largely noting that heavily armoured troops were more affected [8–10]. It was not until British colonisation that more detailed reports were compiled outlining the dangers of transitioning into a hotter environment [11]. Those most affected by the heat were frequently new arrivals in the summer months, indicating that individuals could acclimatise to hot environments within a few months [11]. While this was still a problem during World War I campaigns in the Middle East [10, 12], by World War II an invested interest in how to prevent or limit these problems scientifically was undertaken in military groups. This interest was brought on by a wave of heat-related injuries sustained both in the field and in training camps [13, 14]. Safety regulations were implemented to adjust carried load and clothing worn and to dictate duration and intensity of activities based on the environmental temperature and humidity [14, 15]. Studies progressed further after World War II, looking to minimise the carried load [16] and provide better clothing for such conditions [17, 18], as well as conducting the first investigations into using heat acclimation and acclimatisation to prepare soldiers for hotter environments by eliciting chronic adaptations [19–22]. Planned acclimatisation was investigated first, allowing soldiers a period of time in a new climate to adapt to the environment [19–21, 23–26], while later developments in heat chambers allowed artificial heat exposure to induce such adaptations [27–30], a process known as heat acclimation (HA). From these investigations cardiovascular strain was determined to be a major limiting factor of performance in the heat [31–33], prompting investigations into hydration status and blood volume regulation mechanisms [34–39]. With technological advancements reducing the time to deploy troops to different climates abroad, it is important that research develops to ensure soldiers are adequately prepared when exposed rapidly to extreme climates. Therefore, the aim of this narrative review is to consider 1) the physiology of exercising or working in ambient heat stress in a military context, and 2) the effectiveness and practicality of strategies for coping in hot environments, with an emphasis on short notice deployments and HA. This review will also highlight the challenges of optimising HA within the military, which may identify and guide future directions for military-specific HA research.

## Physiological response during physical work in the heat

In the heat, blood flow is reduced in splanchnic [40–43], inactive muscle [40] and potentially cerebral [44, 45] circulations, to elevate skin blood flow for cooling [46, 47]. During exercise, active skeletal muscle blood flow must also increase substantially to deliver oxygen and substrates to exercising tissue [31, 48]. Work performed by exercising muscle produces heat, which usually comprises the majority of total heat load, requiring a further increase in skin blood flow to protect thermal homeostasis [49, 50]. Skin blood flow typically increases alongside core temperature, but plateaus when core temperature reaches 38 °C, at 50% of maximal skin blood flow [33, 34, 51]. Exercise is facilitated when cerebral, skin and skeletal muscle perfusion requirements are met [34, 48, 52]. However, cardiovascular demand is exacerbated by increases in exercise intensity that promote blood flow to skeletal muscle (if it has not plateaued), while increases in core or skin temperature elevate blood flow to the cutaneous vasculature [32, 34, 53]. As blood flow and volume is redistributed peripherally, central blood volume declines, which can be exacerbated in long-duration exercise by sweat-induced dehydration [46, 54, 55]. If central blood volume becomes insufficient to support blood flow requirements, the cutaneous and skeletal muscular circulations compete for the limited blood supply [32, 34, 56].

During the early stages of exercise, it appears intensity is maintained by restricting skin blood flow to provide adequate blood supply to exercising skeletal muscle at the cost of reduced cooling, accelerating the rise in core temperature [32–34, 52, 57]. However, prior to termination of exercise in fixed-workload trials, skeletal muscle blood flow is reduced [31]. In self-paced exercise, a reduction in exercise intensity occurs early as an anticipatory response to limit the strain of the competing circulations and allow task completion [49, 58–60]. Although measures of integrated electromyographic activity indicate that motor unit recruitment reduces [49, 61–63], this is not accompanied by a reduction in vascular conductance in the exercising muscle [31, 32]. This indicates that the reduction in skeletal muscle blood flow is due to a drop in central blood pressure caused by exacerbated cardiovascular demand [32], secondary to high temperature of peripheral tissue [31, 64, 65]. It is unknown whether reduced motor unit recruitment occurs alongside or because of high core temperature, or whether other physiological feedback mechanisms play a more substantial role [33, 44, 66, 67].

**Fig. 1:**
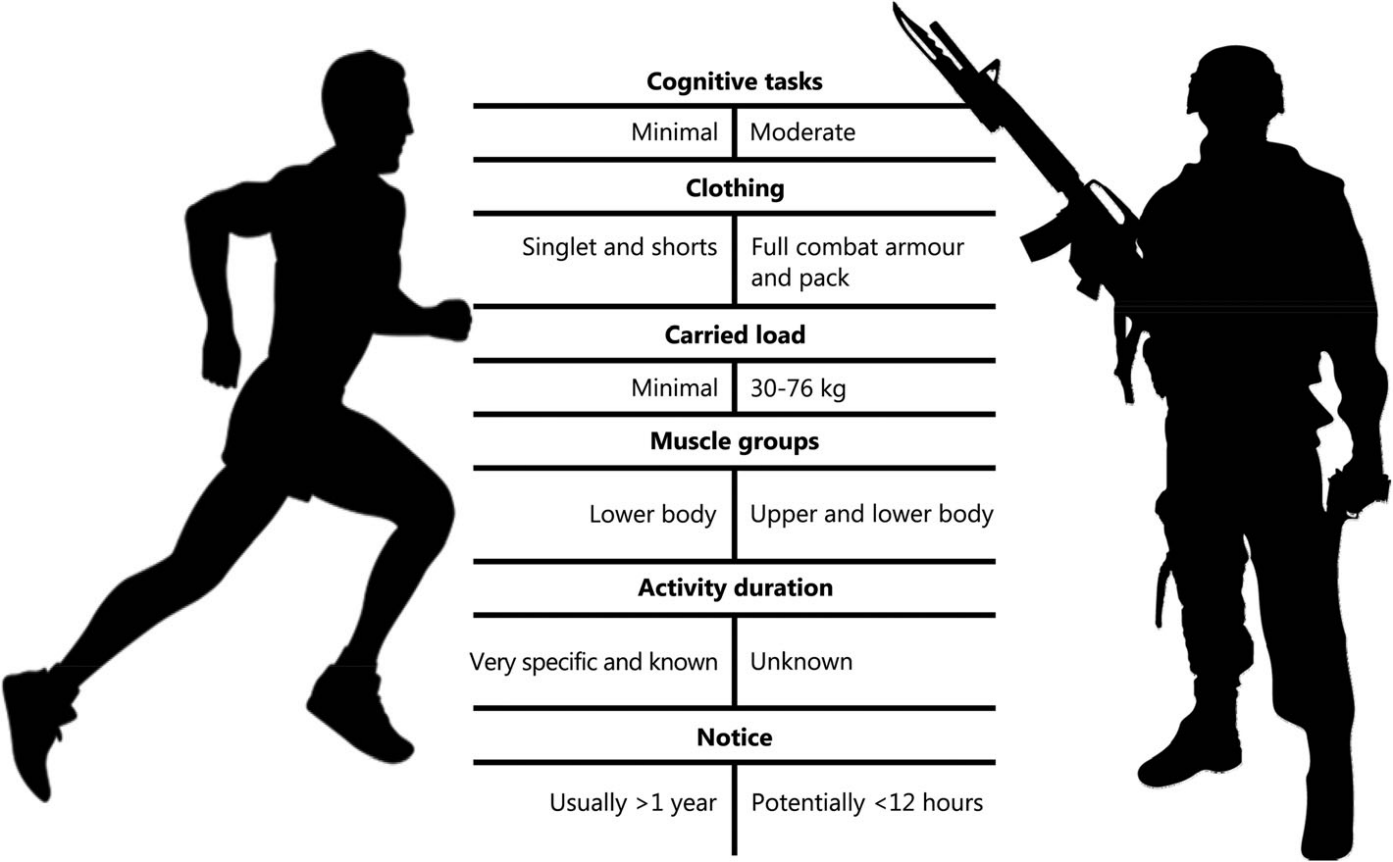
Differences in performance expectations between athletes and soldiers

Much of this research has uncovered physiological processes and mechanisms that have been determined within, and applied to, athletic situations, thereby under-representing military-specific factors that increase and complicate the challenges of exercising in the heat (Fig. 1). For example, protective clothing and equipment reduces skin-to-air contact, impairing convective and evaporative heat loss [47], while also affecting radiative heat gain depending on both the layers and permeability of clothing [68]. In this situation skin blood flow increases in an attempt to dissipate heat, further straining the limited blood supply while achieving little additional heat loss [69]. The combined weight of body armour, webbing and a backpack adds to physiological demand by increasing metabolic cost, elevating skeletal muscle blood flow requirements [70]. Furthermore, soldiers typically have a lower cardiovascular fitness level than the athletes used in the majority of heat research [71–73], so would likely have a lower thermal tolerance [74], even before considering carried loads and restrictive clothing. Altogether, these aspects exaggerate cardiovascular demand, diminish work capacity [75, 76] and predispose soldiers to exertional heat illnesses [10, 77, 78], although these relations remain equivocal [79].

Exertional heat illness occurs in uncompensable heat stress where the metabolic heat production of exercise is unable to be offset by heat exchange with the environment, and exercise intensity is insufficiently downregulated by the individual, leaving them incapacitated [80]. While this often is present in the form of heat exhaustion, extreme cases can cause heat stroke, which can impair central nervous system function [81, 82], cause organ damage [83, 84] and lead to death [12, 14, 85, 86]. Investigations at a basic training facility found 2% of recruits suffered exertional heat illness during summer months [87], while others observed a 40% higher all-cause mortality at a 30-year follow-up in those with prior exertional heat illness [88]. While this could be associated with chronic organ and tissue damage [88], whether the relationship is or is not causal remains to be determined.

## Strategies to combat the effects of heat

Acute heat exposure negatively affects both physical and mental performance, therefore a strategy to maintain performance and health is desirable [89]. Many military operations require relocating to hotter environments for specific missions including disaster relief and unexpected events, before being withdrawn. Deployment notice can sometimes be very short (~ 12 h) allowing minimal, if any, time for heat preparation. These scenarios highlight the importance of strategies to reduce the effects of the heat. Such strategies can occur at the level of the environment, and the level of the individual. For example, the living environment can often be modified in advance to minimise thermoregulatory requirements (i.e. air-conditioned barracks, shaded areas) [15, 90], and aid thermal recovery following exertion (i.e. ice baths, cold water) [82]. Similarly, personal protective equipment should be designed to facilitate heat loss and minimise burdened weight [16, 91].

Several acute strategies can be used at the level of the individual to off-set heat strain, but the unpredictable nature of military tasks and rapidly changing circumstances mean they cannot always be relied upon [92]. Nonetheless, behavioural modification (i.e. shade-seeking, rest) can prevent elevations in core temperature [86], and are often inherent within military guidelines in the form of heat index charts to minimise casualties [93, 94]. This attenuated rise in core temperature can also be achieved with cooling mechanisms, such as ice vests [95] or cold-water immersion [96], used before, during or after exercise. However, ice vests can be uncomfortable, impair body armour function, add to carried weight and requires facilities to generate ice [97, 98]. Similarly, cold-water immersion, the gold-standard method for lowering core temperature [9, 82], requires access to large quantities of cool water. When water supply is limited it should preferentially be used for drinking and hygiene. If facilities are available, consumed water should be chilled [99] and supplemented with electrolytes to prevent hyponatraemia [100] and increase palatability [101].

These strategies are all viable options, but all are limited by being potentially unavailable. Therefore, strategies such as HA that take place prior to deployment can be more controlled and develop adaptations that would be augmented by the acute strategies outlined above.

### Heat acclimation

HA can prevent decrements in both physical and cognitive performance [89], and likely reduces organ damage [102], by altering key underlying physiological variables. It is likely that subcellular adaptations to transport, stress, contractile and metabolic proteins (reviewed elsewhere [103–105]) help lower resting body temperature, enhance heat dissipation and tolerance [59, 106, 107] and potentially reduce metabolic rate [108]. Systemic adaptations also occur with plasma volume expansion increasing skin blood flow and sweat rate which help lower core and skin temperatures. These adaptations allow a higher exercise intensity to be maintained at a given core temperature, or for a lower core temperature to be maintained at a given exercise intensity, thereby improving performance and reducing injury susceptibility [3, 4, 40].

On-site heat acclimatisation over several weeks is regarded as the best-practice methodology to adjust personnel to the environment they will operate in [59, 109]. Even then, arrival in the environment for acclimatisation can pose a challenge as personnel are not adjusted to the heat. Recent research, primarily in athletic contexts, has used HA to induce adaptations comparable to on-site acclimatisation [96, 109]. However, military-specific factors may prevent the direct transferal of athletic findings to military populations (Fig. 1).

For an in-depth review on methods to minimise the effects of heat on military personnel, when ample deployment notice is given, we refer the reader to the recent review of Parsons et al. [89]. However, alternatives are required when notice is noticeably shorter; less than one week and, on occasion, less than one day [96, 109]. Therefore, this section aims to discuss the adaptations that occur as a result of HA, and how they relate to a military environment.

### Physiological adaptations

#### Plasma volume

**Fig. 2:**
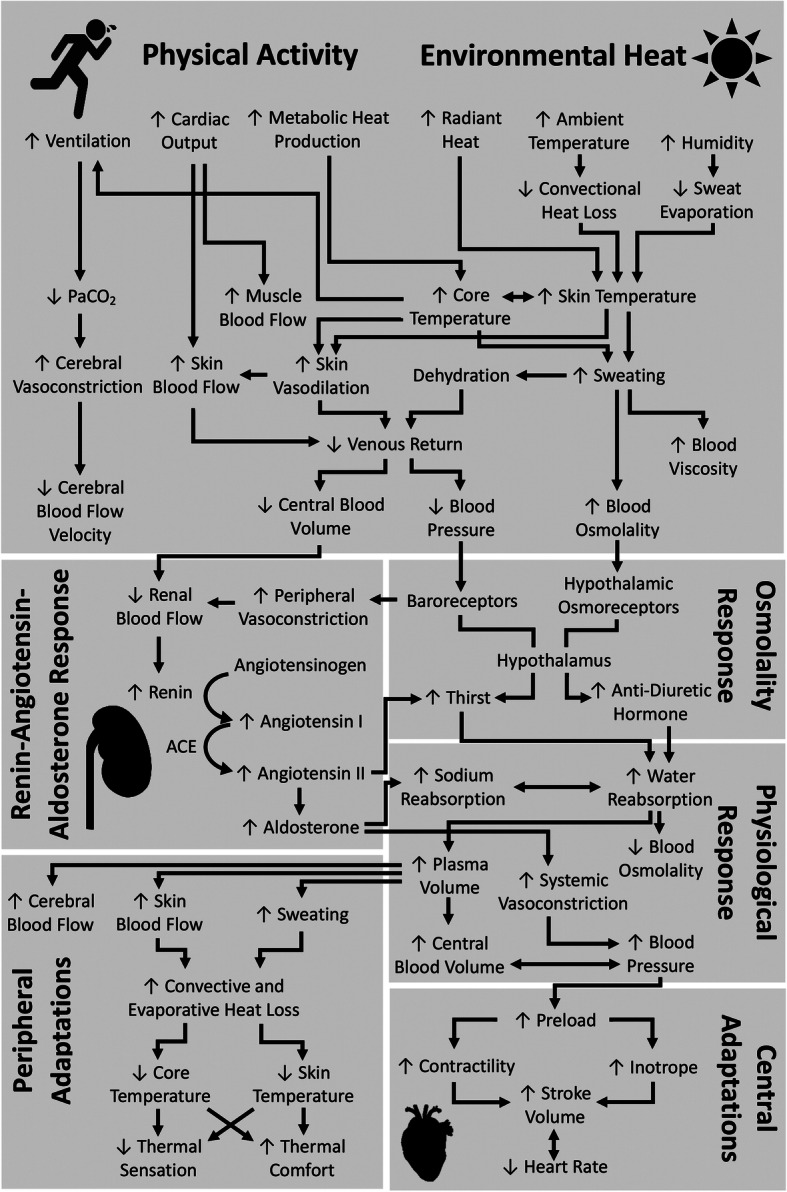
Schematic detailing some of the body’s major responses to exercise in the heat, from the acute responses to the chronic adaptations that occur with repeated exposures

During acute heat exposure plasma volume is redistributed and eventually declines as fluid is lost primarily through sweat, but also respiratory losses, urine and faecal formation [110]. As a result, blood availability for skin and skeletal muscle becomes restricted, which can reduce performance in high-intensity or long duration events by limiting cooling and skeletal muscle blood flow. However, with continuous exercise, low central blood volume, reduced renal blood flow and plasma hyperosmolality stimulate aldosterone and anti-diuretic hormone secretion, upregulating fluid retention mechanisms and stimulating thirst to restore plasma and therefore blood volumes (Fig. 2) [59, 111]. When repeatedly exposed to this stimulus the increase in plasma volume exceeds baseline values, aiding resilience against subsequent exposures. HA programmes have seen plasma volume expansion in as little as two days [112]. Plasma volume increases of more than 20% have been reported [113, 114], although ~ 7% is more common [115–117]. The greater blood volume likely facilitates blood supply to active skeletal muscle and cutaneous circulations simultaneously [54, 59], reducing the cardiovascular burden, therefore increasing the cardiovascular reserve to support performance at higher intensities or for longer durations.

As increasing plasma volume effectively increases the amount of blood that can be distributed throughout the body, studies have explored ways of augmenting the increase seen with HA. For instance, permissive dehydration, to exacerbate the reduction in central blood volume, is hypothesised to increase aldosterone concentration, promote fluid retention and stimulate thirst [117–119]. Garrett et al. [117] found 5 days of HA (involving 90 min cycling per day with core temperature maintained at 38.5 °C in 35 °C, 60% relative humidity (RH)) in an experimental group abstaining from fluid intake, [117] to obtain an 8% mean increase in plasma volume, compared to only a 4% mean increase in controls drinking ad libitum during sessions. Alternatively, consumption of protein supplements immediately post-exercise has been explored [120–122]. These supplements increase plasma albumin content, creating an oncotic gradient to draw fluid into the vascular space to elevate blood volume [120–122]. However, it has not been investigated as to whether supplement-derived plasma volume enhancement leads to improved performance.

#### Heart rate

Elevated blood volume caused by HA enables blood pressure to be maintained in the heat, even with high blood flow demands from skeletal muscle and the cutaneous circulation. While animal studies indicate that over time this could potentially invoke morphological cardiac adaptations that increase ventricular compliance and inotropic state [123, 124], the increased preload helps increase stroke volume [59] allowing heart rate to decrease while maintaining cardiac output [125, 126]. Heart rate is also independently elevated by high core and skin temperatures to increase cutaneous blood flow [127]. During HA, core and skin temperature decline, therefore heart rate is reduced. While lowered heart rate does not improve thermoregulation per se, it indicates a larger cardiac reserve, therefore a reduced cardiovascular strain, making it an optimal outcome for any HA programme. Heart rate can be seen to decline in the first few days of a HA programme, suggesting the changes underlying this occur rapidly, making them relevant to short HA timeframes desired by the military [128–130].

#### Skin blood flow

Skin blood flow translocates thermal energy to the surface, heating the skin to facilitate convective heat loss between the skin and the air, while also facilitating evaporation (and production) of sweat, thereby cooling the blood. With elevated plasma volume, blood flow to cutaneous vasculature increases, facilitating heat dissipation (Fig. 2) [55, 131]. However, while many studies report increased skin blood flow after HA [116, 117, 120, 132] some observe decreases [41], or even no change [40, 118, 132, 133]. These inconsistencies may be due to differences in acute hydration state brought on by water restrictions in some studies. Alternatively, the plateauing of skin blood flow during exercise [34] may increase blood transit time through cutaneous circulations. Therefore, a more complete heat loss can occur, helping to widen the core-to-skin temperature gradient, facilitating heat transfer away from the core [57].

Despite inconsistencies around changes in skin blood flow, mechanistic studies have found HA to increase skin blood flow at a given core temperature in fixed-intensity trials, helping increase the rate of heat dissipation [29, 125, 134–136]. When wearing military clothing any HA-mediated increase in skin blood flow will likely be less effective in promoting heat loss than current research would assume but may still contribute to total heat loss and slow the rise in core temperature. Within this, it is important that soldiers do not compromise the limited skin-air contact that does exist. Certain sunscreens [137, 138] and deodorants [138, 139] can impair both convective and evaporative heat loss more than others, while similar problems may occur when using eye-black or camouflage paint.

#### Sweat rate

Sweating provides the main avenue of heat loss in hot environments [109, 140] and at elevated work-rates, if humidity is low enough to facilitate its evaporation [141]. Hypotonic fluid is secreted from a vast network of eccrine sweat glands located across almost the entire body surface. Sweat output and its distribution show large variability across individuals and body segments, and also varies with exercise intensity, posture, local skin pressure and temperature [142–144], while the cooling (i.e., evaporative) outcome will be further governed by overlying clothing and load carriage patterns [144]. With HA the core temperature threshold for sweating reduces [40], while sweat gland sensitivity heightens [29], thereby increasing the overall sweat response [145]. Furthermore, primate studies reveal that sweat glands undergo hypertrophy [146] and have increased blood supply [147] following HA, likely due to increased skin blood flow, which facilitates sweat production and secretion [148]. By increasing sweat production more heat is lost (assuming low humidity), reducing the rate of rise in core temperature, and enabling exercise at higher intensities or for longer durations (Fig. 2). Additionally, sweat electrolyte content decreases [54, 149], shown by reduced sodium concentration [59, 150, 151] and osmolality [152, 153], which help protect against hyponatraemia [154–156]. Sweating adjustments to HA take the longest to occur [24, 125, 157], but are seen in some short-term protocols [118, 158–164], albeit at lower magnitudes [125]. Interestingly, increases in sweat rate may not be beneficial to military personnel operating in high humidity environments where sweat cannot evaporate, or those wearing protective clothing with low moisture permeability, and in extreme cases those required to wear protective suits [5]. Such clothing can impede or prevent sweat from evaporating, raising the microclimate humidity [165, 166] and causing sweating to occur without heat loss, resulting in dehydration which is detrimental to performance [38, 82, 96, 167, 168]. As dehydration is highly likely to occur in such situations fluid intake is required to minimise negative effects on cognitive [169–171] and physical performance [74, 172, 173], whilst also helping to maintain central blood volume. Advice for hydration has already been well reviewed in current literature [167, 174–176], although for some military units access to water may be limited and those guidelines may require modification. Assuming HA is beneficial, personnel wearing restrictive clothing may benefit from shorter HA protocols that induce a lesser sweat response, but still provide other valuable adaptations (see below).

#### Core temperature

A high core temperature is associated with negative effects on comfort, inflammatory responses [177], organ function [83, 84], descending corticomotor drive [49, 178], as well as performance [40, 44, 49, 179, 180]. Typically, HA evokes a lower resting core temperature [160] and reduces the rate of rise in core temperature due to improved heat dissipation [181]. Increases in sweat rate and convectional heat loss from increased skin blood flow, usually enables a lower core temperature at the same relative intensity as before HA (Fig. 2). In an investigation involving military personnel, Cheung and McLellan [74] found 10 days of HA (10 days of walking at 4.8 km/h at a 3–7% gradient for 1 h in combat clothing in 40 °C, 30% relative humidity over two weeks), to reduce resting core temperature by ~0.2 °C regardless of fitness level, in line with most studies of less than 14 days [125]. A lower resting core temperature increases the heat-sink capacity of the body, enabling more work to be achieved before cooling mechanisms are upregulated [117, 182, 183].

#### Skin temperature

As core temperature decreases with HA, it is important that skin temperature also reduces to ensure the core-to-skin temperature gradient facilitates heat transfer to the periphery [184, 185]. Although rarely seen at rest [183], during exercise, skin temperature lowers with HA [162, 186, 187]. However, in a military context, the influence of clothing minimises skin to air contact, mitigating the rate of cooling. Instead, benefits would likely be seen through the role of skin temperature in the perception of heat. Skin temperature initiates behavioural thermoregulation [188] and has been suggested to majorly contribute to pacing strategies [58, 65]. By lowering skin temperature with HA, perceptual and physiological enhancements help to prolong exercise [173].

### Perceptual changes

Skin temperature [58, 172], heart rate and core temperature all play a role in regulating perceptual responses to heat [189, 190]. As these reduce with HA, exercise in the heat feels easier, shown by improved thermal comfort, thermal sensation and rating of perceived exertion [191–193], especially following active HA [194]. Some short-term HA studies see changes primarily in perceptual outcomes, with negligible improvements to physiological variables [195]. In military settings a combination of peer-pressure and adrenaline can easily overcome perceptual inputs, placing soldiers in physiological danger without being aware of how their body is responding [194].

### Aerobic performance

Endurance performance is impaired in high ambient temperatures [196, 197], by a combination of factors including elevated core and skin temperature [172, 189], reduced central blood volume [46, 54, 55, 65, 198], systemic low-grade inflammation [42, 177] and perceptual responses to heat [96]. Physiological changes that improve heat dissipation mechanisms enable endurance performance in the heat to be improved following HA [125, 199]. A meta-analysis by Tyler et al. [125] showed medium (7–14 days) and long term (> 14 days) HA protocols improved performance by ~20%, while short-term (< 7 days) protocols resulted in improvements of 7%. Improvements are measured either with a time to exhaustion test, or a time-trial test, with time to exhaustion tests showing disproportionally greater improvements [125]. Time to exhaustion tests have been criticised for being invalid for sporting situations compared to a time-trial which integrates perceptual responses through the self-regulation of pace [200, 201]. However, in a military setting they can be just as valid as some military tasks are conducted at fixed intensities.

### Individual variation in heat tolerance

Physiological differences between people causes individual variation in the heat [202], where some perform better than others, despite similar performances in temperate conditions [80, 160, 203, 204]. Those who struggle with heat exposure, and indeed those who have had prior heat illness, should undergo heat-tolerance testing [205, 206]. These individuals may require extra medical attention after deployment, or be closely supervised during additional HA before deployment, to induce protective physiological adaptations that will help minimise heat injury risk [82, 207, 208]. Despite these efforts it is still likely that some soldiers arriving in hot environments will experience adverse effects [10, 12].

There are sub-populations of soldiers that are more prone to heat illness than others. Sex differences likely place females at a thermoregulatory disadvantage, mostly due to anthropometric differences [209, 210]. Furthermore, females sweat less than males [211, 212] which, although potentially minimises dehydration, can reduce heat loss [211]. Accordingly, it has been suggested that females may require additional HA to obtain the same adaptations as males [162, 213]. Unfortunately, limited information exists regarding the effects of menstrual cycle on thermal tolerance and adaptations [214–218] due to the majority of studies in females controlling for this by testing women at the same time-point in their menstrual cycle [161].

Aging also has an effect on thermal tolerance, with older people having higher core temperatures and lower sweat rates, increasing their risk of heat illness [219, 220] if opportunity for behavioural thermoregulation (e.g., lower pacing or resting) is constrained. While age does not appear to affect responsiveness to HA [125], the lower baseline for thermal tolerance in older personnel means they would likely benefit from extended HA protocols [219, 220]. Despite this, studies looking at heat illness incidence rates often find no relationship with age, possibly due to absolute fitness requirements [221, 222].

Fitness is tightly linked with improved performance in the heat [74, 223], with those with a higher fitness level being more economical [224] and having enhanced cardiovascular capacity that helps combat the effects of heat [59]. However, fitness is not a substitute for HA [202] and does not affect the degree of heat adaptation [125]. Finally, there are other important factors impacting on thermal tolerance, and although considered beyond the scope of this review, include medication [225], disease [226, 227], skin coverings such as camouflage paint and tattoos [228], sleep restriction [229], and jet-lag [230]. It is important that group leaders are aware of these factors during deployment so they can assess and monitor each individual and adjust activity levels and exposure (if possible). Furthermore, in the event of a heat illness, additional monitoring is required to safely manage symptoms until appropriate medical attention can be administered [231]. Monitoring technology may enhance this [232–234], but may not be useful in extreme scenarios, emphasising the importance of pre-deployment heat adaptation.

### Cognitive performance

As acute cognitive performance has been reviewed elsewhere, both during exercise [235–237], and in passive (non-exercising) heat stress [238–241] this section will focus on areas relevant both to the military and HA.

Military tasks require a diverse cognitive workload rarely seen in athletic situations, which along with the complex nature of cognitive testing [242], may be why this area of performance has received comparatively little attention. Retrospective military reports indicate that cognitive errors are more common in higher temperatures [241, 243], but these claims are not supported by experimental studies in the wider literature [238, 240, 244, 245]. The discrepancy likely centres around task-specific responses to low-risk, lab-based, cognitive testing [246, 247], and a lack of standardisation in regard to the heat stimulus [239]. Furthermore, many studies have a delay between exercise termination and cognitive testing [92, 248–250], and therefore do not capture cognitive performance during exercise. The recovery period likely enables cognitive function to recover and improve compared to performance during exercise [235, 237], which may make them invalid and potentially misleading for military applications in which critical decision making can occur during exercise.

The effects of HA on cognitive performance are less clear. Studies have shown improvements in reaction time [92] and correct responses to rapid visual processing [92], no effect on visual inattention [246] and simple motor performance [92, 246] while time perception has improved [251] or worsened [246] in different studies. Certain adaptations to HA may aid cerebral function by way of constraining hyperthermia, maintaining cerebral blood flow or lessening discomfort and therefore distraction effects. Although cerebral blood flow increases with exercise intensities up to 60% V̇O_2max_, it declines at higher intensities [252–254]; a relationship likely further influenced by exercise duration and exacerbated by high body temperature [45, 255]. Therefore, increased plasma volume may be thought to improve cognitive function through increased cerebral blood flow at these intensities, for reasons mentioned above. However, alterations in cerebral blood flow do not appear to affect cognitive performance. Cerebral autoregulation enables cerebral blood flow to be maintained despite changes in mean arterial blood pressure brought on by both exercise and heat reducing cerebral blood flow [57, 126, 253]. Brain oxygen, glucose and lactate uptake are not related to reduced cerebral blood flow, but do decline at very high intensity exercise [253, 256]. The continued uptake of nutrients may explain why when manipulating cerebral blood flow with hypercapnia there are minimal changes to cognitive performance [257]. Other mechanistic findings indicate that cerebral neural activation [257] or alterations in cerebral metabolism [258] may play a larger role. The lack of understanding as to why cognitive performance appears affected, and may be improved, is an aspect of HA that is yet to be properly investigated. While it is hard to recreate military decision making in the manner it would be encountered in the field, it is important to use tasks of a similar nature to better understand the foundational cognitive processes being undertaken and how they might be affected both by heat and with HA. Recent studies have offered new insights using brain imaging technology to analyse cognitive function in the heat, even showing that head cooling may help overcome the negative impact of hyperthermia [259]. Future studies should extend this by adding exercise to the paradigm, to develop a greater understanding of what causes impairments to cognitive function in the heat.

### How to achieve heat acclimation

The efficacy of several different HA protocols has been reported in the literature. Many factors, such as the heat modality, ambient conditions, frequency of sessions, number of sessions and duration of each session can be adjusted to influence the physiological response to heat and exercise. Furthermore, while these factors have often been chosen or adapted to produce the best outcomes in sporting applications, they can be impractical for military contexts. For example, most HA protocols involve exercising in a heat chamber, allowing environmental conditions to replicate that of a desired environment [118, 128, 160, 161, 183, 260]. However, these facilities are often hard to access and cannot accommodate large numbers of participants. Within each session most studies use low or moderate intensity exercise (45–65% V̇O_2max_) [2, 116, 158, 159, 186, 261, 262], with sessions lasting at least one hour [117, 118, 128, 132, 152, 158, 161, 183, 187, 261]. For the military this would mean adding extra exercise sessions, which may impair other training [177, 263], and could result in overtraining [264].

**Fig. 3:**
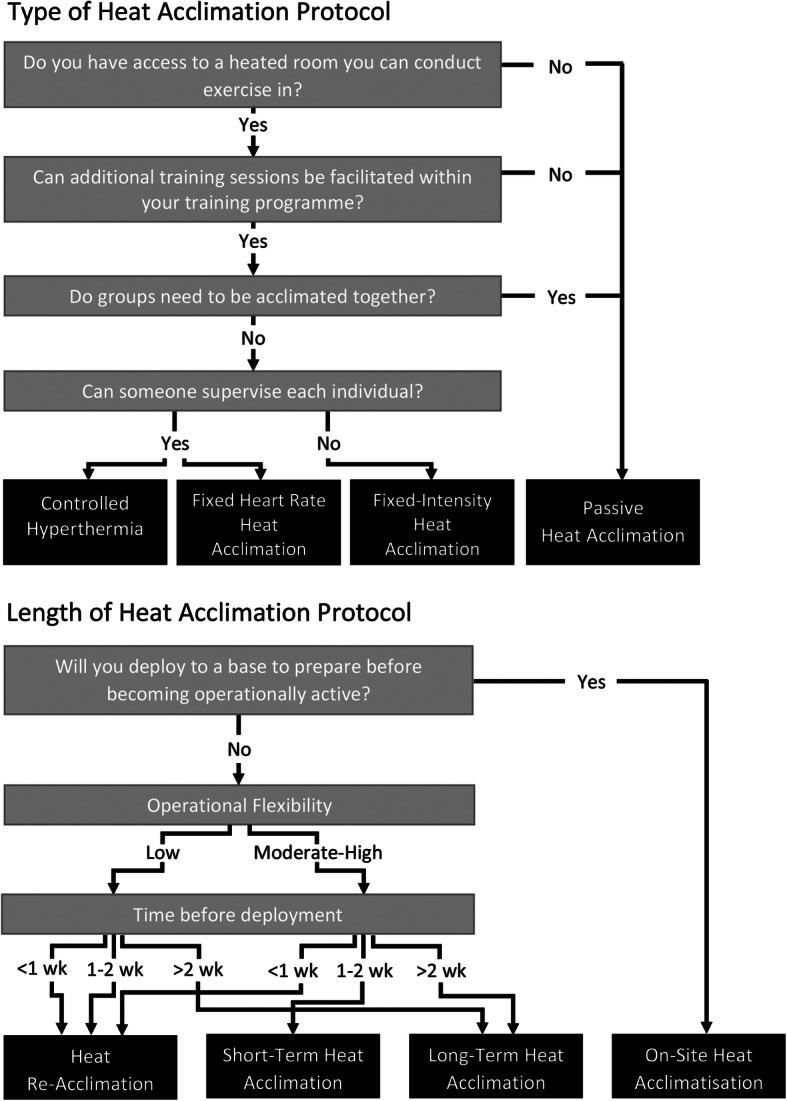
Flow-diagrams to determine the most appropriate form of heat acclimation, and how long that heat acclimation protocol should be, prior to deployment. Groups are defined as >5 personnel. Operational flexibility is the ability to change the activities done during the operation (i.e. ability to stop and rest or change the objective). wk: weeks

Recent athletic HA studies have made an effort to maintain the thermal strain during exercise across a HA regime using the controlled hyperthermia technique, i.e. intensity is adjusted regularly to ensure core temperature is maintained at 38.5 °C during exercise sessions [114, 117, 118, 128, 152, 161, 162, 183, 186, 260, 262, 265, 266], but this requires additional equipment and vigilant monitoring of participants using invasive equipment (Fig. 3). Furthermore, typical HA programmes take two weeks to see meaningful changes in sweat responses [109, 152, 163, 164, 178, 267], which does not suit military groups with short deployment notices.

#### Short-term heat acclimation

Recent literature has focused on optimising short-term (< 7 days) HA [2, 41, 117, 118, 120, 128, 158–162, 177, 183, 195, 260, 268–272] to minimise the barriers present in HA and prevent potential interference with higher priority training objectives [158, 195, 263]. However, this method adds additional training sessions, which may promote fatigue and overtraining, while also requiring access to facilities, such as heat chambers [183, 186]. The reduced programme length also limits the magnitude of adaptations [125]. While a lack of increase in sweat rate might limit dehydration, this may occur at the cost of other adaptations, and would soon develop upon deployment [160]. While short-term HA may be practical for athletic groups (and some military situations), the reduced magnitude of changes makes it less desirable for groups deploying with ample notice, and the length of the programme is likely incompatible for short-notice deployment units (Fig. 3). For deployments of slightly longer notice, it might serve as a primer for more chronic adaptations to develop during deployment.

#### Passive heat acclimation methods

Passive heat exposure modalities, such as saunas or hot-water immersion facilities, allow groups of people to acclimate at once (Fig. 3), and can be installed easily. Passive HA may alleviate concerns that essential training may be impaired by additional sessions being conducted in the heat (Fig*.* 3), although to what extent normal training can be affected is currently unclear. Passive heat methods have typically been employed post-exercise, allowing exercise to elevate core temperature that can be maintained or further increased by passive heat [273–275], while there may also be benefits to having exercise metabolites in the circulation during passive heating [276]. The following sections consider passive heating methods currently in use and the evidence surrounding their physiological and functional effectiveness.

##### Sauna

Saunas are a hot-dry (65 °C - 110 °C, 10–30% relative humidity) room designed to induce cardiovascular strain and a sweat response [277]. Increases in peripheral blood distribution help invoke cardiovascular and hormonal adaptations that are beneficial for coping in the heat (Fig. 2). Scoon et al. [273] reported four 30-min post-exercise saunas (90 °C) per week for three weeks increased plasma volume by 7%, while improving time to exhaustion by 32%. Supporting this, Stanley et al. [278] found a plasma volume increase of 18% after four 30-min post-exercise sauna exposures (87 °C, 11% RH). The larger increase could be due to sessions occurring on consecutive days, although further comparison between studies is limited by the lack of a performance test.

##### Hot-water immersion

Hot-water immersion works similarly to sauna and has also been used to maintain an elevated core body temperature after exercise. For six consecutive days Zurawlew et al. [274] had an experimental group bathe in 40 °C water for 40 min following training, allowing for an additional ~ 1 °C rise in rectal temperature, compared to controls bathed in 34 °C water. In a heated (33 °C, 40% RH) 5-km running time-trial the intervention group improved 4.9%, linked to lower core and skin temperature, and an earlier sweat onset. Similar findings were observed in a subsequent study, by the same research group, which also saw reductions in end-exercising heart rate and V̇O_2_, and no significant change in plasma volume [275]. Brazaitis et al. [279] produced similar findings using immersion in 44 °C water up to the waist for 45 min on alternating days over a two-week period, without prior exercise. Although no performance test was conducted, this resulted in lower core temperature (↓ 0.3 °C), heart rate (↓ 12 beat/min) and psychological strain, and an increased sweat rate (↑ 40%) during hot-water immersion on day 14 when compared to day 1 [279].

In summary, both sauna and hot water immersion provide a heat exposure stimulus without requiring additional exercise, which may benefit military groups with heavy training schedules. These two passive heating modalities are yet to be directly compared in a HA context, so it is unclear whether they offer unique adaptations that may help before travelling to stressful environments. Furthermore, no study had compared either method to a more traditional, exercising HA programme, so it is unclear what differences exist in maximal adaptive capacity as well as the rates of adaptation each offer. Further investigations are required to evaluate the performance of both passive heating methods and to optimise their ability to induce heat adaptations.

##### Heat re-acclimation

One method that could enable rapid HA in military personnel is re-acclimation. This topic has recently been thoroughly reviewed elsewhere [280], and so will only be discussed briefly here. Heat re-acclimation occurs following a period away from heat after completing an initial HA programme. Here, a few HA sessions are used in attempt to restore, or maintain, heat adaptations which otherwise begin to decay [59]. As studies in this area are relatively limited, results vary, with some studies finding a day of HA is lost with every two days without heat stimuli [178], while a single HA session every 5 days can sustain adaptations [59, 281]. Within a military context, where deployment time can be unknown, re-acclimation could maintain a heat-acclimated state for a long period of time. Despite this area of research still being in its infancy, the potential to regain the adaptations of a previously completed heat-acclimation regime in a very short space of time (Fig. 3), or to continuously maintain them is promising. Within the military, maintaining these adaptations after acclimatising on deployment or during training in a warmer climate are both convenient and feasible. This would allow heat-acclimated soldiers to deploy to hotter climates at very short notice with minimised heat-related performance impairments.

### Recommendations

On-site acclimatisation in the operational environment allows all-day exposure to the environmental conditions that will be worked in, and therefore is often the most desirable way to adjust to the heat. However, this is not always possible, and indeed deployment to this environment must also be considered. In more temperate environments where soldiers typically train, HA is an effective way to induce physiological adaptations to protect the body against thermal disturbances to homeostasis. Traditionally heat chambers are used for HA [54, 125], although this does require additional training sessions and can make it hard to acclimate large groups of people [263]. However, if these limitations are irrelevant then use of heat chambers for HA is well supported [125]. Whether or not supervision (both by person and technical equipment) is available to support these sessions dictates whether controlled hyperthermia or fixed-intensity exercise should be conducted (Fig*.* 3).

Controlled hyperthermia is currently considered the best-practice method for HA [152, 186], although it does require invasive and expensive equipment to provide real-time measures of core temperature, and because of this has often been overlooked in favour of fixed-intensity protocols [89]. Alternatively, when such facilities are unavailable, or additional training is undesireable, passive heat acclimation can provide an effective alternative (Fig. 3). While further studies are required to determine the best use of this approach, the results from recent studies are promising, demonstrating both performance enhancing and protective adaptations [274, 278, 282].

When considering the length of the heat acclimation protocol it is important to consider how HA integrates with other training requirements. This may impact the length of the HA protocol as conducting sessions on consecutive days may become impractical [263]. Furthermore, the overall load and fatigue state of soldiers during HA phases also requires attention as arriving at the deployed destination with in an over trained state will render adaptations meaningless [6, 222]. If operational flexibility is low (i.e. mission objectives are fixed and must be completed within a precise timeframe) soldier fatigue state becomes even more desirable than usual for HA adaptations to be as complete as possible as there is limited opportunity for behavioural adjustments to the heat [58]. Therefore, the use of a long-term HA protocol to generate these adaptations is ideal, although if notice before deployment is short re-acclimation would be more suitable (Fig*.* 3). The re-acclimation would require having already conducted a long-term HA protocol, and then maintaining those adaptations for a prolonged period, to hold an elevated baseline heat tolerance. As such a strategy exists in a unique situation of long-term planning but short notice, this would likely only be used in special units that know they could be called upon at any time to deploy into different climates. If operational flexibility is moderate-high, then heat acclimation prior to deployment would likely act as a primer to minimise the effects of heat immediately upon arrival and provide a baseline heat tolerance that would be enhanced in the coming days (Fig. 3). Regardless, if ample notice is given prior to deployment, it makes sense to obtain maximally beneficial adaptations, if other training allows it.

## Conclusions

Both physical and cognitive performance are impaired during exercise in the heat. With HA underlying physiological adaptations enhance thermoregulatory and cardiovascular function, which are responsible for at least part of the improved performance in the heat. While HA studies to date inform strategies for athletes preparing for competition in the heat, there is minimal consideration of military specific factors such as restrictive clothing, carried loads, large groups being acclimated, or short deployment notice. For military units that might expect to deploy at short notice (< 1 day) the options regarding heat acclimation are limited, beyond proactive planning to maintain heat adaptations for a prolonged and unspecified period. However, this approach has received limited attention in the literature due to this unique set of circumstances. Therefore, further investigations are required to optimise HA for military application. Specifically, the identification of effective, practical and feasible methods of HA, or re-acclimation, which can be undertaken by large groups of military personnel at short notice to prepare for deployment to hot environments.

## Data Availability

Not applicable.

## Abbreviations

HA: Heat acclimation
RH: Relative humidity

## References

[1] Brotherhood J (2008). Heat stress and strain in exercise and sport. J Sci Med Sport.

[2] Charlot K, Tardo-Dino PE, Buchet JF, Koulmann N, Bourdon S, Lepetit B (2017). Short-term, low-volume training improves heat acclimatization in an operational context. Front Physiol.

[3] Hargreaves M (2008). Physiological limits to exercise performance in the heat. J Sci Med Sport.

[4] Junge N, Jørgensen R, Flouris AD, Nybo L (2016). Prolonged self-paced exercise in the heat-environmental factors affecting performance. Temperature..

[5] Sawka M, Wenger CB, Young AJ, Pandolf KB, Research IOMUCOMN, Marriott B (1993). Physiological responses to exercise in the heat. Nutritional needs in hot environments: applications for military personnel in field operations.

[6] Schmit C, Duffield R, Hausswirth C, Brisswalter J, Le Meur Y (2018). Optimizing heat acclimation for endurance athletes: high- vs low-intensity training. Int J Sports Physiol Perform.

[7] Stubblefield Z, Cleary M, Garvey S, Eberman L (2006). Effects of active hyperthermia on cognitive performance. Proceedings of the fifth annual college of education research conference: section on allied health professions.

[8] Goldman RF, Pandoff KB, Re (2002). Introduction to heat-related problems in military operations. Medical aspects of harsh environments.

[9] Casa DJ, Mcdermott BP, Lee EC, Yeargin SW, Armstrong LE, Maresh CM (2007). Cold water immersion: the gold standard for exertional heatstroke treatment. Exerc Sport Sci Rev.

[10] Bricknell M (1995). Heat illness - a review of military experience (part 1). J R Army Med.

[11] Lind J (2015). An essay on diseases incidental to Europeans in hot climates with the method of preventing their fatal consequences, 1771.

[12] Willcox WH (1920). The nature, prevention, and treatment of heat hyperpyrexia: the clinical aspect. Br Med J.

[13] Cook EL (1955). Epidemiological approach to heat trauma. Mil Med.

[14] Schickele E (1947). Environment and fatal heat stroke; an analysis of 157 cases occurring in the Army in the U. S. during world war II. Mil Surg.

[15] Yaglou CP, Minard D (1957). Control of heat casualties at military training centers. AMA Arch Ind Health.

[16] Knapik JJ, Ang P, Meiselman H, Johnson W, Kirk J, Bensel C (1997). Soldier performance and strenuous road marching: influence of load mass and load distribution. Mil Med.

[17] Joy RJ, Goldman RF (1968). A method of relating physiology and military performance. A study of some effects of vapor barrier clothing in a hot climate. Mil Med.

[18] Gonzales RR (1986). Biophysical and physiological integration of proper clothing for exercise.

[19] Lind AR, Bass DE (1963). Optimal exposure time for development of acclimatization to heat. Fed Proc.

[20] Fox RH, Goldsmith R, Kidd DJ, Lewis HE (1963). Blood flow and other thermoregulatory changes with acclimatization to heat. J Physiol.

[21] Ladell WS (1951). Assessment of group acclimatization to heat and humidity. J Physiol.

[22] Adolph EF (1969). Physiology of man in the desert: Hafner publishing company.

[23] Wyndham CH, Strydom NB, Morrison JF, Du Toit FD, Kraan JG (1954). Responses of unacclimatized men under stress of heat and work. J Appl Physiol.

[24] Fox RH, Goldsmith R, Hampton IF, Lewis HE (1964). The nature of the increase in sweating capacity produced by heat acclimatization. J Physiol.

[25] Strydom NB, Wyndham CH, Williams CG, Morrison JF, Bredell GA, Benade AJ (1966). Acclimatization to humid heat and the role of physical conditioning. J Appl Physiol.

[26] Piwonka RW, Robinson S (1967). Acclimatization of highly trained men to work in severe heat. J Appl Physiol.

[27] Gonzalez RR, Pandolf KB, Gagge AP (1974). Heat acclimation and decline in sweating during humidity transients. J Appl Physiol.

[28] Fein J, Haymes E, Buskirk E (1975). Effects of daily and intermittent exposures on heat acclimation of women. Int J Biometeorol.

[29] Roberts MF, Wenger CB, Stolwijk JA, Nadel ER (1977). Skin blood flow and sweating changes following exercise training and heat acclimation. J Appl Physiol Respir Environ Exerc Physiol.

[30] Shvartz E, Bhattacharya A, Sperinde SJ, Brock PJ, Sciaraffa D, Van Beaumont W (1979). Sweating responses during heat acclimation and moderate conditioning. J Appl Physiol Respir Environ Exerc Physiol.

[31] Gonzalez-Alonso J, Calbet JA (2003). Reductions in systemic and skeletal muscle blood flow and oxygen delivery limit maximal aerobic capacity in humans. Circ J.

[32] Gonzalez-Alonso J, Calbet JA, Nielsen B (1998). Muscle blood flow is reduced with dehydration during prolonged exercise in humans. J Physiol.

[33] Gonzalez-Alonso J, Teller C, Andersen SL, Jensen FB, Hyldig T, Nielsen B (1999). Influence of body temperature on the development of fatigue during prolonged exercise in the heat. J Appl Physiol (1985).

[34] Gonzalez-Alonso J, Crandall CG, Johnson JM (2008). The cardiovascular challenge of exercising in the heat. J Physiol.

[35] Nadel ER, Fortney SM, Wenger CB (1980). Effect of hydration state of circulatory and thermal regulations. J Appl Physiol Respir Environ Exerc Physiol.

[36] Francesconi RP, Sawka MN, Pandolf KB (1983). Hypohydration and heat acclimation: plasma renin and aldosterone during exercise. J Appl Physiol Respir Environ Exerc Physiol.

[37] Cadarette BS, Sawka MN, Toner MM, Pandolf KB (1984). Aerobic fitness and the hypohydration response to exercise-heat stress. Aviat Space Environ Med.

[38] Sawka MN, Young AJ, Latzka WA, Neufer PD, Quigley MD, Pandolf KB (1992). Human tolerance to heat strain during exercise: influence of hydration. J Appl Physiol (1985).

[39] Gonzalez-Alonso J, Mora-Rodriguez R, Below PR, Coyle EF (1995). Dehydration reduces cardiac output and increases systemic and cutaneous vascular resistance during exercise. J Appl Physiol (1985).

[40] Nielsen B, Hales JR, Strange S, Christensen NJ, Warberg J, Saltin B (1993). Human circulatory and thermoregulatory adaptations with heat acclimation and exercise in a hot, dry environment. J Physiol.

[41] Chen TI, Tsai PH, Lin JH, Lee NY, Liang MT (2013). Effect of short-term heat acclimation on endurance time and skin blood flow in trained athletes. J Sports Med.

[42] Selkirk GA, Mclellan TM, Wright HE, Rhind SG (2008). Mild endotoxemia, NF-kappaB translocation, and cytokine increase during exertional heat stress in trained and untrained individuals. Am J Phys Regul Integr Comp Phys.

[43] Crandall CG, Wilson TE, Marving J, Vogelsang TW, Kjaer A, Hesse B (2008). Effects of passive heating on central blood volume and ventricular dimensions in humans. J Physiol.

[44] Nybo L, Nielsen B (2001). Hyperthermia and central fatigue during prolonged exercise in humans. J Appl Physiol.

[45] Nybo L, Nielsen B (2017). Middle cerebral artery blood velocity is reduced with hyperthermia during prolonged exercise in humans. J Physiol.

[46] Charkoudian N (2016). Human thermoregulation from the autonomic perspective. Auton Neurosci.

[47] Cramer M, Gagnon D, Crandall CG, Jay O (2016). Does attenuated skin blood flow lower sweat rate and the critical environmental limit for heat balance during severe heat exposure?. Exp Physiol.

[48] Lee KWJ, Kuah LF, Wasan PS, Lee T, Tan PL, Seko A (2017). Short term training in a cool vs. warm environment on aerobic performance in a warm and humid condition. J Sci Med Sport.

[49] Tucker R, Rauch L, Harley YX, Noakes TD (2004). Impaired exercise performance in the heat is associated with an anticipatory reduction in skeletal muscle recruitment. Eur J Phys.

[50] González-Alonso J, Quistorff B, Krustrup P, Bangsbo J, Saltin B. Heat production in human skeletal muscle at the onset of intense dynamic exercise. J Physiol 2000;524(Pt 2):603–615. 10.1111/j.1469-7793.2000.00603.x PMC2269891 10766936

[51] Racinais S, Cocking S, Periard JD (2017). Sports and environmental temperature: from warming-up to heating-up. Temperature (Austin).

[52] Savard GK, Nielsen B, Laszczynska J, Larsen BE, Saltin B (1988). Muscle blood flow is not reduced in humans during moderate exercise and heat stress. J Appl Physiol (1985).

[53] Greaney JL, Kenney WL, Alexander LM (2016). Sympathetic regulation during thermal stress in human aging and disease. Auton Neurosci.

[54] Taylor NA (2000). Principles and practices of heat adaptation. J Hum Environ Syst.

[55] Tripathi A, Mack GW, Nadel ER (1990). Cutaneous vascular reflexes during exercise in the heat. Med Sci Sports Exerc.

[56] Kenefick R (2017). Acute physiological responses during exercise in the heat. J Sci Med Sport.

[57] Kenney WL, Stanhewicz AE, Bruning RS, Alexander LM (2014). Blood pressure regulation III: what happens when one system must serve two masters: temperature and pressure regulation?. Eur J Appl Physiol.

[58] Schlader ZJ, Simmons SE, Stannard SR, Mundel T (2011). Skin temperature as a thermal controller of exercise intensity. Eur J Appl Physiol.

[59] Periard JD, Travers GJ, Racinais S, Sawka MN (2016). Cardiovascular adaptations supporting human exercise-heat acclimation. Auton Neurosci.

[60] Tatterson A, Hahn A, Martini D, Febbraio M (2000). Effects of heat stress on physiological responses and exercise performance in elite cyclists. J Sci Med Sport.

[61] Tyler CJ, Sunderland C, Cheung SS (2015). The effect of cooling prior to and during exercise on exercise performance and capacity in the heat: a meta-analysis. Br J Sports Med.

[62] Wingfield GL, Gale R, Minett GM, Marino FE, Skein M (2016). The effect of high versus low intensity heat acclimation on performance and neuromuscular responses. J Therm Biol.

[63] Reilly T, Drust B, Gregson W (2006). Thermoregulation in elite athletes. Curr Opin Clin Nutr Metab Care.

[64] Cotter JD, Sleivert GG, Roberts WS, Febbraio MA (2001). Effect of pre-cooling, with and without thigh cooling, on strain and endurance exercise performance in the heat. Comp Biochem Physiol A Mol Integr Physiol.

[65] Kenefick RW, Cheuvront SN, Palombo LJ, Ely BR, Sawka MN (2010). Skin temperature modifies the impact of hypohydration on aerobic performance. J Appl Physiol (1985).

[66] Brink-Elfegoun T, Kaijser L, Gustafsson T, Ekblom B (2007). Maximal oxygen uptake is not limited by a central nervous system governor. J Appl Physiol (1985).

[67] Inzlicht M, Marcora SM (2016). The central governor model of exercise regulation teaches us precious little about the nature of mental fatigue and self-control failure. Front Psychol.

[68] Brode P, Kuklane K, Candas V, Den Hartog EA, Griefahn B, Holmer I (2010). Heat gain from thermal radiation through protective clothing with different insulation, reflectivity and vapour permeability. Int J Occup Saf Ergon.

[69] Fogarty AL, Armstrong KA, Gordon CJ, Groeller H, Woods BF, Stocks JM (2004). Cardiovascular and thermal consequences of protective clothing: a comparison of clothed and unclothed states. Ergonomics..

[70] Puthoff ML, Darter BJ, Nielsen DH, Yack HJ (2006). The effect of weighted vest walking on metabolic responses and ground reaction forces. Med Sci Sports Exerc.

[71] Bedno SA, Urban N, Boivin MR, Cowan DN (2014). Fitness, obesity and risk of heat illness among army trainees. Occup Med.

[72] Knapik JJ, Sharp MA, Steelman RA (2017). Secular trends in the physical fitness of United States Army recruits on entry to service, 1975-2013. J Strength Cond Res.

[73] Campos LCB, Campos FAD, Bezerra TAR, Pellegrinotti ÍL (2017). Effects of 12 weeks of physical training on body composition and physical fitness in military recruits. Int J Exerc Sci.

[74] Cheung SS, Mclellan TM (1998). Heat acclimation, aerobic fitness, and hydration effects on tolerance during uncompensable heat stress. J Appl Physiol (1985).

[75] Tomes C, Orr RM, Pope R (2017). The impact of body armor on physical performance of law enforcement personnel: a systematic review. Ann Occup Environ Med.

[76] Johnson RF, Knapik JJ, Merullo DJ (1995). Symptoms during load carrying: effects of mass and load distribution during a 20-km road march. Percept Mot Skills.

[77] Carter R, Cheuvront SN, Williams JO, Kolka MA, Stephenson LA, Sawka MN (2005). Epidemiology of hospitalizations and deaths from heat illness in soldiers. Med Sci Sports Exerc.

[78] Phinney LT, Gardner JW, Kark JA, Wenger CB (2001). Long-term follow-up after exertional heat illness during recruit training. Med Sci Sports Exerc.

[79] Stacey MJ, Parsons IT, Woods DR, Taylor PN, Ross D, J Brett S (2015). Susceptibility to exertional heat illness and hospitalisation risk in UK military personnel. BMJ Open Sport Exerc.

[80] Stacey M, Woods D, Ross D, Wilson D (2014). Heat illness in military populations: asking the right questions for research. J R Army Med Corps.

[81] Epstein Y, Druyan A, Heled Y (2012). Heat injury prevention--a military perspective. J Strength Cond Res.

[82] Casa DJ, Armstrong LE, Kenny GP, O’connor FG, Huggins RA (2012). Exertional heat stroke: new concepts regarding cause and care. Curr Sports Med Rep.

[83] Goforth CW, Kazman JB (2015). Exertional heat stroke in navy and marine personnel: a hot topic. Crit Care Nurse.

[84] Sagui E, Beighau S, Jouvion A, Trichereau J, Cornet D, Berthelot RC (2017). Thermoregulatory response to exercise after exertional heat stroke. Mil Med.

[85] Cheuvront SN, Carter R, Castellani JW, Sawka MN (2005). Hypohydration impairs endurance exercise performance in temperate but not cold air. J Appl Physiol.

[86] Rav-Acha M, Hadad E, Epstein Y, Heled Y, Moran DS (2004). Fatal exertional heat stroke: a case series. Am J Med Sci.

[87] Gardner JW, Kark JA, Karnei K, Sanborn JS, Gastaldo E, Burr P (1996). Risk factors predicting exertional heat illness in male marine corps recruits. Med Sci Sports Exerc.

[88] Wallace RF, Kriebel D, Punnett L, Wegman DH, Amoroso PJ (2007). Prior heat illness hospitalization and risk of early death. Environ Res.

[89] Parsons IT, Stacey MJ, Woods DR (2019). Heat adaptation in military personnel: mitigating risk, maximizing performance. Front Physiol.

[90] Budd GM (2008). Wet-bulb globe temperature (WBGT) - its history and its limitations. J Sci Med Sport.

[91] Adams P, Slocum A, Keyserling W (1994). A model for protective clothing effects on performance. Int J Cloth Sci Technol.

[92] Radakovic SS, Maric J, Surbatovic M, Radjen S, Stefanova E, Stankovic N (2007). Effects of acclimation on cognitive performance in soldiers during exertional heat stress. Mil Med.

[93] Blanchard L, Santee W (2008). Comparison of USARIEM heat strain decision aid, mobile heat stress monitor, and existing army guidelines for warm weather training.

[94] Buller MJ, Welles AP, Stower J, Desantis C, Margolis L, Karis AJ (2011). Thermal-work strain during marine rifle squad operations in Afghanistan (march 2010).

[95] Barr D, Gregson W, Sutton L, Reilly T (2009). A practical cooling strategy for reducing the physiological strain associated with firefighting activity in the heat. Ergonomics..

[96] Racinais S, Alonso JM, Coutts AJ, Flouris AD, Girard O, González-Alonso J (2015). Consensus recommendations on training and competing in the heat. Sports Med.

[97] Kenny GP, Schissler AR, Stapleton J, Piamonte M, Binder K, Lynn A (2011). Ice cooling vest on tolerance for exercise under uncompensable heat stress. J Occup Environ Hyg.

[98] Bongers CC, Hopman MT, Eijsvogels TM (2017). Cooling interventions for athletes: an overview of effectiveness, physiological mechanisms, and practical considerations. Temperature (Austin).

[99] Lee JKW, Yeo ZW, Nio AQX, Koh ACH, Teo YS, Goh LF (2013). Cold drink attenuates heat strain during work-rest cycles. Int J Sports Med.

[100] Sawka MN, Montain SJ (2000). Fluid and electrolyte supplementation for exercise heat stress. Am J Clin Nutr.

[101] Burdon CA, Johnson NA, Chapman PG, O'connor HT (2012). Influence of beverage temperature on palatability and fluid ingestion during endurance exercise: a systematic review. Int J Sport Nutr Exerc Metab.

[102] Omassoli J, Hill NE, Woods DR, Delves SK, Fallowfield JL, Brett SJ (2019). Variation in renal responses to exercise in the heat with progressive acclimatisation. J Sci Med Sport.

[103] Muga A, Moro F (2008). Thermal adaptation of heat shock proteins. Curr Protein Pept Sci.

[104] Horowitz M (2016). Epigenetics and cytoprotection with heat acclimation. J Appl Physiol.

[105] Horowitz M, Robinson SD (2007). Heat shock proteins and the heat shock response during hyperthermia and its modulation by altered physiological conditions. Prog Brain Res.

[106] Periard JD, Racinais S, Sawka MN (2015). Adaptations and mechanisms of human heat acclimation: applications for competitive athletes and sports. Scand J Med Sci Sports.

[107] Taylor NA (2014). Human heat adaptation. Compr Physiol.

[108] Horowitz M (2001). Heat acclimation: phenotypic plasticity and cues to the underlying molecular mechanisms. J Therm Biol.

[109] Bergeron MF, Bahr R, Bartsch P, Bourdon L, Calbet JA, Carlsen KH (2012). International Olympic Committee consensus statement on thermoregulatory and altitude challenges for high-level athletes. Br J Sports Med.

[110] Maughan RJ, Shirreffs SM, Leiper JB (2007). Errors in the estimation of hydration status from changes in body mass. J Sports Sci.

[111] Akerman AP, Tipton M, Minson CT, Cotter JD (2016). Heat stress and dehydration in adapting for performance: good, bad, both, or neither?. Temperature..

[112] Senay LC, Mitchell D, Wyndham CH (1976). Acclimatization in a hot, humid environment: body fluid adjustments. J Appl Physiol.

[113] Costa RJ, Teixeira A, Rama L, Swancott AJ, Hardy LD, Lee B (2013). Water and sodium intake habits and status of ultra-endurance runners during a multi-stage ultra-marathon conducted in a hot ambient environment: an observational field based study. Nutr J.

[114] Beaudin AE, Clegg ME, Walsh ML, White MD (2009). Adaptation of exercise ventilation during an actively-induced hyperthermia following passive heat acclimation. Am J Phys Regul Integr Comp Phys.

[115] Buchheit M, Voss SC, Nybo L, Mohr M, Racinais S (2011). Physiological and performance adaptations to an in-season soccer camp in the heat: associations with heart rate and heart rate variability. Scand J Med Sci Sports.

[116] Fujii N, Honda Y, Ogawa T, Tsuji B, Kondo N, Koga S (2012). Short-term exercise-heat acclimation enhances skin vasodilation but not hyperthermic hyperpnea in humans exercising in a hot environment. Eur J Appl Physiol.

[117] Garrett AT, Goosens NG, Rehrer NJ, Patterson MJ, Harrison J, Sammut I (2014). Short-term heat acclimation is effective and may be enhanced rather than impaired by dehydration. Am J Hum Biol.

[118] Neal RA, Corbett J, Massey HC, Tipton MJ (2016). Effect of short-term heat acclimation with permissive dehydration on thermoregulation and temperate exercise performance. Scand J Med Sci Sports.

[119] Akerman AP, Lucas SJE, Katare R, Cotter JD (2017). Heat and dehydration additively enhance cardiovascular outcomes following orthostatically-stressful calisthenics exercise. Front Physiol.

[120] Goto M, Okazaki K, Kamijo Y, Ikegawa S, Masuki S, Miyagawa K (2010). Protein and carbohydrate supplementation during 5-day aerobic training enhanced plasma volume expansion and thermoregulatory adaptation in young men. J Appl Physiol.

[121] Okazaki K, Hayase H, Ichinose T, Mitono H, Doi T, Nose H (2009). Protein and carbohydrate supplementation after exercise increases plasma volume and albumin content in older and young men. J Appl Physiol (1985).

[122] Okazaki K, Ichinose T, Mitono H, Chen M, Masuki S, Endoh H (2009). Impact of protein and carbohydrate supplementation on plasma volume expansion and thermoregulatory adaptation by aerobic training in older men. J Appl Physiol (1985).

[123] Heled Y, Peled A, Yanovich R, Shargal E, Pilz-Burstein R, Epstein Y (2012). Heat acclimation and performance in hypoxic conditions. Aviat Space Environ Med.

[124] Horowitz M (2003). Matching the heart to heat-induced circulatory load: heat-acclimatory responses. Physiology..

[125] Tyler CJ, Reeve T, Hodges GJ, Cheung SS (2016). The effects of heat adaptation on physiology, perception and exercise performance in the heat: a meta-analysis. Sports Med.

[126] Periard JD, Cramer MN, Chapman PG, Caillaud C, Thompson MW (2011). Cardiovascular strain impairs prolonged self-paced exercise in the heat. Exp Physiol.

[127] Chou TH, Allen JR, Hahn D, Leary BK, Coyle EF (2018). Cardiovascular responses to exercise when increasing skin temperature with narrowing of the core-to-skin temperature gradient. J Appl Physiol (1985).

[128] Garrett AT, Goosens NG, Rehrer NJ, Patterson MJ, Cotter JD (2009). Induction and decay of short-term heat acclimation. Eur J Appl Physiol.

[129] Pandolf KB (1998). Time course of heat acclimation and its decay. Int J Sports Med.

[130] Faulkner SJ, Stephen M, Dale E, Paul S (2016). Review of human acclimatisation to heat or cold.

[131] Nielsen B, Savard G, Richter EA, Hargreaves M, Saltin B (1990). Muscle blood flow and muscle metabolism during exercise and heat stress. J Appl Physiol (1985).

[132] Best S, Thompson M, Caillaud C, Holvik L, Fatseas G, Tammam A (2014). Exercise-heat acclimation in young and older trained cyclists. J Sci Med Sport.

[133] Regan JM, Macfarlane DJ, Taylor NA (1996). An evaluation of the role of skin temperature during heat adaptation. Acta Physiol Scand.

[134] Lorenzo S, Minson CT (2010). Heat acclimation improves cutaneous vascular function and sweating in trained cyclists. J Appl Physiol (1985).

[135] Yamazaki F, Hamasaki K (2003). Heat acclimation increases skin vasodilation and sweating but not cardiac baroreflex responses in heat-stressed humans. J Appl Physiol (1985).

[136] Ravanelli N, Coombs GB, Imbeault P, Jay O (2019). Thermoregulatory adaptations with progressive heat acclimation are predominantly evident in uncompensable, but not compensable, conditions. J Appl Physiol (1985).

[137] House J, Breed M, Cotter J, Lucas S, Mundel T (2013). Sunscreen use reduces sweat evaporation but not production. Environmental ergonomics. Queenstown.

[138] Aburto-Corona J, Aragon-Vargas L (2016). Sunscreen use and sweat production in men and women. J Athl Train.

[139] Quatrale R, Coble D, Stoner K, Felger C (1981). The mechanism of antiperspirant action by aluminum salts. II. Histological observations of human eccrine sweat glands inhibited by aluminum chlorohydrate. J Soc Cosmet Chem.

[140] Adams W, Fox R, Fry A, Macdonald I (1975). Thermoregulation during marathon running in cool, moderate, and hot environments. J Appl Physiol.

[141] Gavin T (2003). Clothing and thermoregulation during exercise. Sports Med.

[142] Kondo N, Takano S, Aoki K, Shibasaki M, Tominaga H, Inoue Y (1998). Regional differences in the effect of exercise intensity on thermoregulatory sweating and cutaneous vasodilation. Acta Physiol Scand.

[143] Nishiyama T, Sugenoya J, Takaaki M, Iwase S, Mano T (2001). Irregular activation of individual sweat glands in human sole observed by a videomicroscopy. Auton Neurosci.

[144] Taylor NA, Machado-Moreira CA (2013). Regional variations in transepidermal water loss, eccrine sweat gland density, sweat secretion rates and electrolyte composition in resting and exercising humans. Extreme Physiol Med.

[145] Sawka MN, Leon LR, Montain SJ, Sonna LA (2011). Integrated physiological mechanisms of exercise performance, adaptation, and maladaptation to heat stress. Comp Physiol.

[146] Sato F, Owen M, Matthes R, Sato K, Gisolfi CV (1990). Functional and morphological changes in the eccrine sweat gland with heat acclimation. J Appl Physiol (1985).

[147] Okuda N, Kanai M, Watar N, Ohara K. Morphological changes of the eccrine sweat glands of Japanese monkey after heat acclimation: the mechanisms of peripheral adaptation. In: Szelenyi Z, Szekely M, editors. Contributions to thermal physiology. Budapest: Pergamon Press; 1980. p. 293–6.

[148] Sato K, Sato F (1983). Individual variations in structure and function of human eccrine sweat gland. Am J Phys.

[149] Schmit C, Le Meur Y, Duffield R, Robach P, Oussedik N, Coutts AJ (2015). Heat-acclimatization and pre-cooling: a further boost for endurance performance?. Scand J Med Sci Sports.

[150] Stacey MJ, Woods DR, Brett SJ, Britland SE, Fallowfield JL, Allsopp AJ (2018). Heat acclimatization blunts copeptin responses to hypertonicity from dehydrating exercise in humans. Phys Rep.

[151] Kirby CR, Convertino VA (1986). Plasma aldosterone and sweat sodium concentrations after exercise and heat acclimation. J Appl Physiol.

[152] Patterson MJ, Stocks JM, Taylor NA (2014). Whole-body fluid distribution in humans during dehydration and recovery, before and after humid-heat acclimation induced using controlled hyperthermia. Acta Physiol (Oxford).

[153] Marshall H, Campbell S, Roberts C, Nimmo M (2007). Human physiological and heat shock protein 72 adaptations during the initial phase of humid-heat acclimation. J Therm Biol.

[154] Winger JM, Dugas JP, Dugas LR (2011). Beliefs about hydration and physiology drive drinking behaviours in runners. Br J Sports Med.

[155] Oh RC, Malave B, Chaltry JD (2018). Collapse in the heat - from overhydration to the emergency room - three cases of exercise-associated hyponatremia associated with exertional heat illness. Mil Med.

[156] O'brien KK, Montain SJ, Corr WP, Sawka MN, Knapik JJ, Craig SC (2001). Hyponatremia associated with overhydration in U.S. Army trainees. Mil Med.

[157] Armstrong LE, Maresh CM (1991). The induction and decay of heat acclimatisation in trained athletes. Sports Med.

[158] Casadio JR, Kilding AE, Siegel R, Cotter JD, Laursen PB (2016). Periodizing heat acclimation in elite laser sailors preparing for a world championship event in hot conditions. Temperature..

[159] Dileo TD, Powell JB, Kang HK, Roberge RJ, Coca A, Kim JH (2016). Effect of short-term heat acclimation training on kinetics of lactate removal following maximal exercise. J Sports Med Phys Fitness.

[160] Karlsen A, Nybo L, Nørgaard SJ, Jensen MV, Bonne T, Racinais S (2015). Time course of natural heat acclimatization in well-trained cyclists during a 2-week training camp in the heat. Scand J Med Sci Sports.

[161] Mee JA, Gibson OR, Doust J, Maxwell NS (2015). A comparison of males and females' temporal patterning to short- and long-term heat acclimation. Scand J Med Sci Sports.

[162] Mee JA, Peters S, Doust JH, Maxwell NS (2018). Sauna exposure immediately prior to short-term heat acclimation accelerates phenotypic adaptation in females. J Sci Med Sport.

[163] Patterson MJ, Stocks JM, Taylor NA (2004). Humid heat acclimation does not elicit a preferential sweat redistribution toward the limbs. Am J Phys Regul Integr Comp Phys.

[164] Poirier MP, Gagnon D, Friesen BJ, Hardcastle SG, Kenny GP (2015). Whole-body heat exchange during heat acclimation and its decay. Med Sci Sports Exerc.

[165] Ueda H, Inoue Y, Matsudaira M, Araki T, Havenith G (2006). Regional microclimate humidity of clothing during light work as a result of the interaction between local sweat production and ventilation. Int J Cloth Sci Technol.

[166] Taylor NA (2015). Overwhelming physiological regulation through personal protection. J Strength Cond Res.

[167] Cotter JD, Thornton SN, Lee JK, Laursen PB (2014). Are we being drowned in hydration advice? Thirsty for more?. Extreme Physiol Med.

[168] Fortney SM, Wenger CB, Bove JR, Nadel ER (1984). Effect of hyperosmolality on control of blood flow and sweating. J Appl Physiol Respir Environ Exerc Physiol.

[169] Adan A (2012). Cognitive performance and dehydration. J Am Coll Nutr.

[170] Grandjean AC, Grandjean NR (2007). Dehydration and cognitive performance. J Am Coll Nutr.

[171] Lieberman HR (2007). Hydration and cognition: a critical review and recommendations for future research. J Am Coll Nutr.

[172] Sawka MN, Cheuvront SN, Kenefick RW (2012). High skin temperature and hypohydration impair aerobic performance. Exp Physiol.

[173] Cheuvront SN, Kenefick RW, Montain SJ, Sawka MN (2010). Mechanisms of aerobic performance impairment with heat stress and dehydration. J Appl Physiol.

[174] Koulmann N, Banzet S, Bigard AX (2003). Physical activity in the heat: physiology of hydration recommendations. Med Trop (Mars).

[175] Mccartney D, Desbrow B, Irwin C (2017). The effect of fluid intake following dehydration on subsequent athletic and cognitive performance: a systematic review and meta-analysis. J Sport Med.

[176] Shirreffs SM (2009). Symposium on performance, exercise and health hydration, fluids and performance. Proc Nutr Soc.

[177] Guy JH, Pyne DB, Deakin GB, Miller CM, Edwards AM (2016). Acclimation training improves endurance cycling performance in the heat without inducing endotoxemia. Front Physiol.

[178] Garrett AT, Rehrer NJ, Patterson MJ (2011). Induction and decay of short-term heat acclimation in moderately and highly trained athletes. Sports Med.

[179] Marino FE, Mbambo Z, Kortekaas E, Wilson G, Lambert MI, Noakes TD (2000). Advantages of smaller body mass during distance running in warm, humid environments. Pflugers Arch.

[180] Galloway SD, Maughan RJ (1997). Effects of ambient temperature on the capacity to perform prolonged cycle exercise in man. Med Sci Sports Exerc.

[181] Daanen HA, Jonkman AG, Layden JD, Linnane DM, Weller AS (2011). Optimising the acquisition and retention of heat acclimation. Int J Sports Med.

[182] Shen D, Zhu N (2015). Influence of the temperature and relative humidity on human heat acclimatization during training in extremely hot environments. Build Environ.

[183] James CA, Richardson AJ, Watt PW, Willmott AG, Gibson OR, Maxwell NS (2017). Short-term heat acclimation improves the determinants of endurance performance and 5-km running performance in the heat. Appl Physiol Nutr Metab.

[184] Smolander J, Saalo J, Korhonen O (1991). Effect of work load on cutaneous vascular response to exercise. J Appl Physiol (1985).

[185] Gisolfi C, Robinson S (1969). Relations between physical training, acclimatization, and heat tolerance. J Appl Physiol.

[186] Gibson OR, Mee JA, Tuttle JA, Taylor L, Watt PW, Maxwell NS (2015). Isothermic and fixed intensity heat acclimation methods induce similar heat adaptation following short and long-term timescales. J Therm Biol.

[187] Racinais S, Buchheit M, Bilsborough J, Bourdon PC, Cordy J, Coutts AJ (2014). Physiological and performance responses to a training camp in the heat in professional australian football players. Sport Physiol Perform.

[188] Schlader ZJ, Perry BG, Jusoh MR, Hodges LD, Stannard SR, Mundel T (2013). Human temperature regulation when given the opportunity to behave. Eur J Appl Physiol.

[189] Periard JD, Racinais S, Knez WL, Herrera CP, Christian RJ, Girard O (2014). Thermal, physiological and perceptual strain mediate alterations in match-play tennis under heat stress. Br J Sports Med.

[190] Flouris AD, Schlader ZJ (2015). Human behavioral thermoregulation during exercise in the heat. Scand J Med Sci Sports.

[191] Chalmers S, Esterman A, Eston R, Bowering KJ, Norton K (2014). Short-term heat acclimation training improves physical performance: a systematic review, and exploration of physiological adaptations and application for team sports. Sports Med.

[192] Saat M, Sirisinghe RG, Singh R, Tochihara Y (2005). Decay of heat acclimation during exercise in cold and exposure to cold environment. Eur J Appl Physiol.

[193] Lee BJ, Miller A, James RS, Thake CD (2016). Cross acclimation between heat and hypoxia: heat acclimation improves cellular tolerance and exercise performance in acute normobaric hypoxia. Front Physiol.

[194] Malgoyre A, Tardo-Dino PE, Koulmann N, Lepetit B, Jousseaume L, Charlot K (2018). Uncoupling psychological from physiological markers of heat acclimatization in a military context. J Therm Biol.

[195] Kelly M, Gastin PB, Dwyer DB, Sostaric S, Snow RJ (2016). Short duration heat acclimation in australian football players. J Sports Sci Med.

[196] Ely MR, Cheuvront SN, Roberts WO, Montain SJ (2007). Impact of weather on marathon-running performance. Med Sci Sports Exerc.

[197] El-Helou N, Tafflet M, Berthelot G, Tolaini J, Marc A, Guillaume M (2012). Impact of environmental parameters on marathon running performance. PLoS One.

[198] Macdougall JD, Reddan WG, Layton CR, Dempsey JA (1974). Effects of metabolic hyperthermia on performance during heavy prolonged exercise. J Appl Physiol.

[199] Keiser S, Fluck D, Huppin F, Stravs A, Hilty MP, Lundby C (2015). Heat training increases exercise capacity in hot but not in temperate conditions: a mechanistic counter-balanced cross-over study. Am J Physiol Heart Circ Physiol.

[200] Atkinson G, Peacock O, St Clair Gibson A, Tucker R (2007). Distribution of power output during cycling: impact and mechanisms. Sports Med.

[201] Laursen PB, Francis GT, Abbiss CR, Newton MJ, Nosaka K (2007). Reliability of time-to-exhaustion versus time-trial running tests in runners. Med Sci Sports Exerc.

[202] Corbett J, Rendell RA, Massey HC, Costello JT, Tipton MJ (2018). Inter-individual variation in the adaptive response to heat acclimation. J Therm Biol.

[203] Kampmann B, Brode P, Schutte M, Griefahn B (2008). Lowering of resting core temperature during acclimation is influenced by exercise stimulus. Eur J Appl Physiol.

[204] Mcdermott BP, Casa DJ, Yeargin SW, Ganio MS, Armstrong LE, Maresh CM (2007). Recovery and return to activity following exertional heat stroke: considerations for the sports medicine staff. J Sport Rehabil.

[205] Moran DS, Erlich T, Epstein Y (2007). The heat tolerance test: an efficient screening tool for evaluating susceptibility to heat. J Sport Rehabil.

[206] Schermann H, Heled Y, Fleischmann C, Ketko I, Schiffmann N, Epstein Y (2018). The validity of the heat tolerance test in prediction of recurrent exertional heat illness events. J Sci Med Sport.

[207] Taylor N, Patterson M, Regan J, Amos D (1997). Heat acclimation procedures: preparation for humid heat exposure.

[208] Druyan A, Ketko I, Yanovich R, Epstein Y, Heled Y (2013). Refining the distinction between heat tolerant and intolerant individuals during a heat tolerance test. J Therm Biol.

[209] Corbett J, Wright J, Tipton MJ. Sex differences in response to exercise heat stress in the context of the military environment. BMJ Mil Health. 2020;jramc-2019-001253. 10.1136/jramc-2019-001253. 10.1136/jramc-2019-001253 32094215

[210] Kazman JB, Purvis DL, Heled Y, Lisman P, Atias D, Van Arsdale S, et al. Women and exertional heat illness: identification of gender specific risk factors. US Army Med Dep J. 2015; PMID: 26101907. 26101907

[211] Sawka MN, Toner MM, Francesconi RP, Pandolf KB (1983). Hypohydration and exercise: effects of heat acclimation, gender, and environment. J Appl Physiol.

[212] Shapiro Y, Pandolf KB, Avellini BA, Pimental NA, Goldman RF (1980). Physiological responses of men and women to humid and dry heat. J Appl Physiol Respir Environ Exerc Physiol.

[213] Mee J, Peters S, Doust J, Maxwell N (2015). Restricted sweat evaporation preceding short term heat acclimation accelerates adaption in females. Extrem Physiol Med.

[214] Armstrong LE, Maresh CM, Keith NR, Elliott TA, Vanheest JL, Scheett TP (2005). Heat acclimation and physical training adaptations of young women using different contraceptive hormones. Am J Physiol Endocrinol Metab.

[215] Carpenter AJ, Nunneley SA (1988). Endogenous hormones subtly alter women's response to heat stress. J Appl Physiol (1985).

[216] Inoue Y, Tanaka Y, Omori K, Kuwahara T, Ogura Y, Ueda H (2005). Sex- and menstrual cycle-related differences in sweating and cutaneous blood flow in response to passive heat exposure. Eur J Appl Physiol.

[217] Kolka MA, Stephenson LA (1989). Control of sweating during the human menstrual cycle. Eur J Appl Physiol Occup Physiol.

[218] Tenaglia SA, Mclellan TM, Klentrou PP (1999). Influence of menstrual cycle and oral contraceptives on tolerance to uncompensable heat stress. Eur J Appl Physiol Occup Physiol.

[219] Kenney WL, Hodgson JL (1987). Heat tolerance, thermoregulation and ageing. Sports Med.

[220] Wagner JA, Robinson S, Tzankoff SP, Marino RP (1972). Heat tolerance and acclimatization to work in the heat in relation to age. J Appl Physiol.

[221] King MA, Ward MD, Mayer TA, Plamper ML, Madsen CM, Cheuvront SN (2019). Influence of prior illness on exertional heat stroke presentation and outcome. PLoS One.

[222] Li Q, Sun R, Liu S, Lyu H, Wang H, Hu Q (2018). Effect of heat acclimatization training on inflammatory reaction and multiple organ dysfunction syndrome in patients with exertional heat stroke. Zhonghua Wei Zhong Bing Ji Jiu Yi Xue.

[223] Pandolf KB, Burse RL, Goldman RF (1977). Role of physical fitness in heat acclimatisation, decay and reinduction. Ergonomics..

[224] Smoljanić J, Morris NB, Dervis S, Jay O. Running economy, not aerobic fitness, independently alters thermoregulatory responses during treadmill running. J Appl Physiol (1985). 2014:1451–9. 10.1152/japplphysiol.00665.2014 PMC4269685 25301893

[225] Ramphal-Naley L (2012). Screening for heat stress in workers and athletes. Proc (Baylor Univ Med Cent).

[226] Armstrong LE, Casa DJ, Millard-Stafford M, Moran DS, Pyne SW, Roberts WO (2007). American College of Sports Medicine position stand. Exertional heat illness during training and competition. Med Sci Sports Exerc.

[227] Nelson DA, Deuster PA, O'connor FG, Kurina LM (2018). Sickle cell trait and heat injury among US Army soldiers. Am J Epidemiol.

[228] Luetkemeier MJ, Hanisko JM, Aho KM (2017). Skin tattoos alter sweat rate and Na^+^ concentration. Med Sci Sports Exerc.

[229] Gregory LB, Gerald PK, Thomas JB, Donald BH, Robert ES (1987). Effects of continuous operations (CONCOPS) on soldier and unit performance: review of the literature and strategies for sustaining the soldier in CONCOPS.

[230] Monk TH (1991). Sleep and circadian rhythms. Exp Gerontol.

[231] Donoghue M, Sinclair M, Bates G (2000). Heat exhaustion in a deep underground metalliferous mine. Occup Environ Med.

[232] Pham S, Yeap D, Escalera G, Basu R, Wu X, Kenyon NJ, et al. Wearable sensor system to monitor physical activity and the physiological effects of heat exposure. Sensors (Basel). 2020;20(3). 10.3390/s20030855 PMC7039288 32041097

[233] Buller MJ, Welles AP, Friedl KE (2018). Wearable physiological monitoring for human thermal-work strain optimization. J Appl Physiol.

[234] Tharion W, Buller M, Potter A, Karis A, Goetz V, Hoyt R (2013). Acceptability and usability of an ambulatory health monitoring system for use by military personnel. IIE Trans Occup Ergon Hum Factors.

[235] Lambourne K, Tomporowski P (2010). The effect of exercise-induced arousal on cognitive task performance: a meta-regression analysis. Brain Res.

[236] Tomporowski PD (2003). Effects of acute bouts of exercise on cognition. Acta Psychol.

[237] Chang YK, Labban JD, Gapin JI, Etnier JL (2012). The effects of acute exercise on cognitive performance: a meta-analysis. Brain Res.

[238] Hancock PA, Vasmatzidis I (2003). Effects of heat stress on cognitive performance: the current state of knowledge. Int J Hyperth.

[239] Gaoua N (2010). Cognitive function in hot environments: a question of methodology. Scand J Med Sci Sports.

[240] Schmit C, Hausswirth C, Le Meur Y, Duffield R (2017). Cognitive functioning and heat strain: performance responses and protective strategies. Sports Med.

[241] Martin K, Mcleod E, Periard J, Rattray B, Keegan R, Pyne DB (2019). The impact of environmental stress on cognitive performance: a systematic review. Hum Factors.

[242] Lieberman HR, Bathalon GP, Falco CM, Morgan CA, Niro PJ, Tharion WJ (2005). The fog of war: decrements in cognitive performance and mood associated with combat-like stress. Aviat Space Environ Med.

[243] Froom P, Caine Y, Shochat I, Ribak J (1993). Heat stress and helicopter pilot errors. J Occup Med.

[244] Bhattacharyya D, Pal M, Chatterjee T, Majumdar D (2017). Effect of load carriage and natural terrain conditions on cognitive performance in desert environments. Physiol Behav.

[245] Hancock PA, Ross JM, Szalma JL (2007). A meta-analysis of performance response under thermal stressors. Hum Factors.

[246] Patterson M, Taylor N, Amos D (1998). Physical work and cognitive function during acute heat exposure before and after heat acclimation.

[247] Gaoua N, Racinais S, Grantham J, El Massioui F (2011). Alterations in cognitive performance during passive hyperthermia are task dependent. Int J Hyperth.

[248] Jimenez-Pavon D, Romeo J, Cervantes-Borunda M, Ortega F, Ruiz JR, Espana-Romero V (2011). Effects of a running bout in the heat on cognitive performance. J Exerc Sci Fit.

[249] Cian C, Barraud PA, Melin B, Raphel C (2001). Effects of fluid ingestion on cognitive function after heat stress or exercise-induced dehydration. Int J Psychophysiol.

[250] Grego F, Vallier JM, Collardeau M, Rousseu C, Cremieux J, Brisswalter J (2005). Influence of exercise duration and hydration status on cognitive function during prolonged cycling exercise. Int J Sports Med.

[251] Tamm M, Jakobson A, Havik M, Timpmann S, Burk A, Ööpik V (2015). Effects of heat acclimation on time perception. Int J Psychophysiol.

[252] Moraine JJ, Lamotte M, Berre J, Niset G, Leduc A, Naeije R (1993). Relationship of middle cerebral artery blood flow velocity to intensity during dynamic exercise in normal subjects. Eur J Appl Physiol Occup Physiol.

[253] Ogoh S, Ainslie PN (2009). Cerebral blood flow during exercise: mechanisms of regulation. J Appl Physiol (1985).

[254] Hellstrom G, Fischer-Colbrie W, Wahlgren NG, Jogestrand T (1996). Carotid artery blood flow and middle cerebral artery blood flow velocity during physical exercise. J Appl Physiol (1985).

[255] Nybo L, Møller K, Volianitis S, Nielsen B, Secher NH (2002). Effects of hyperthermia on cerebral blood flow and metabolism during prolonged exercise in humans. J Appl Physiol.

[256] Ide K, Schmalbruch IK, Quistorff B, Horn A, Secher NH (2000). Lactate, glucose and O_2_ uptake in human brain during recovery from maximal exercise. J Physiol.

[257] Ogoh S, Tsukamoto H, Hirasawa A, Hasegawa H, Hirose N, Hashimoto T (2014). The effect of changes in cerebral blood flow on cognitive function during exercise. Phys Rep.

[258] Ogoh S (2017). Relationship between cognitive function and regulation of cerebral blood flow. J Physiol Sci.

[259] Xue Y, Li L, Qian S, Liu K, Zhou XJ, Li B (2018). The effects of head-cooling on brain function during passive hyperthermia: an fMRI study. Int J Hyperth.

[260] Garrett AT, Creasy R, Rehrer NJ, Patterson MJ, Cotter JD (2012). Effectiveness of short-term heat acclimation for highly trained athletes. Eur J Appl Physiol.

[261] Cotter JD, Patterson MJ, Taylor NA (1997). Sweat distribution before and after repeated heat exposure. Eur J Appl Physiol Occup Physiol.

[262] Weller AS, Linnane DM, Jonkman AG, Daanen HA (2007). Quantification of the decay and re-induction of heat acclimation in dry-heat following 12 and 26 days without exposure to heat stress. Eur J Appl Physiol.

[263] Casadio J, Kilding A, Cotter J, Laursen P (2017). From lab to real world: heat acclimation considerations for elite athletes. Sports Med.

[264] Walker A, Mckune A, Ferguson S, Pyne DB, Rattray B (2016). Chronic occupational exposures can influence the rate of PTSD and depressive disorders in first responders and military personnel. Extreme Physiol Med.

[265] Magalhaes Fde C, Amorim FT, Passos RL, Fonseca MA, Oliveira KP, Lima MR (2010). Heat and exercise acclimation increases intracellular levels of Hsp72 and inhibits exercise-induced increase in intracellular and plasma Hsp72 in humans. Cell Stress Chaperones.

[266] Ruddock AD, Thompson SW, Hudson SA, James CA, Gibson OR, Mee JA (2016). Combined active and passive heat exposure induced heat acclimation in a soccer referee before 2014 FIFA world cup. SpringerPlus..

[267] Voltaire B, Galy O, Coste O, Recinais S, Callis A, Blonc S (2002). Effect of fourteen days of acclimatization on athletic performance in tropical climate. Can J Appl Physiol.

[268] Brade C, Dawson B, Wallman K (2013). Effect of precooling and acclimation on repeat-sprint performance in heat. J Sports Sci.

[269] Petersen CJ, Portus MR, Pyne DB, Dawson BT, Cramer MN, Kellett AD (2010). Partial heat acclimation in cricketers using a 4-day high intensity cycling protocol. Int J Sports Physiol Perform.

[270] Sunderland C, Morris JG, Nevill ME (2008). A heat acclimation protocol for team sports. Br J Sports Med.

[271] Walker SM, Dawson B, Ackland TR (2001). Performance enhancement in rally car drivers via heat acclimation and race simulation. Comp Biochem Physiol A Mol Integr Physiol.

[272] Willmott AG, Gibson OR, Hayes M, Maxwell NS (2016). The effects of single versus twice daily short term heat acclimation on heat strain and 3000 m running performance in hot, humid conditions. J Therm Biol.

[273] Scoon GS, Hopkins WG, Mayhew S, Cotter JD (2007). Effect of post-exercise sauna bathing on the endurance performance of competitive male runners. J Sci Med Sport.

[274] Zurawlew MJ, Walsh NP, Fortes MB, Potter C (2016). Post-exercise hot water immersion induces heat acclimation and improves endurance exercise performance in the heat. Scand J Med Sci Sports.

[275] Zurawlew MJ, Michael JA, Walsh NP. Heat acclimation by post-exercise hot water immersion in the morning reduces thermal strain during morning and afternoon exercise-heat-stress. Int J Sport Physio Perform. 2018. 10.1123/ijspp.2017-0620. 10.1123/ijspp.2017-0620 29745780

[276] Kissling LS, Akerman AP, Cotter JD (2019). Heat-induced hypervolemia: does the mode of acclimation matter and what are the implications for performance at Tokyo 2020?. Temperature (Austin).

[277] Hannuksela ML, Ellahham S (2001). Benefits and risks of sauna bathing. Am J Med.

[278] Stanley J, Halliday A, D'auria S, Buchheit M, Leicht AS (2015). Effect of sauna-based heat acclimation on plasma volume and heart rate variability. Eur J Appl Physiol.

[279] Brazaitis M, Skurvydas A (2010). Heat acclimation does not reduce the impact of hyperthermia on central fatigue. Eur J Appl Physiol.

[280] Daanen HM, Racinais S, Periard JD (2018). Heat acclimation decay and re-induction: a systematic review and meta-analysis. Sports Med.

[281] Pryor JL, Pryor RR (2019). Intermittent exercise-heat exposures and intense physical activity sustain heat acclimation adaptations. J Sci Med Sport.

[282] Zurawlew MJ, Mee JA, Walsh NP (2018). Post-exercise hot water immersion elicits heat acclimation adaptations in endurance trained and recreationally active individuals. Front Physiol.

